# TMEM241 is a UDP-N-acetylglucosamine transporter required for M6P modification of NPC2 and cholesterol transport

**DOI:** 10.1016/j.jlr.2023.100465

**Published:** 2023-10-27

**Authors:** Nan Zhao, Gang Deng, Pei-Xin Yuan, Ya-Fen Zhang, Lu-Yi Jiang, Xiaolu Zhao, Bao-Liang Song

**Affiliations:** The Institute for Advanced Studies, Hubei Key Laboratory of Cell Homeostasis, College of Life Sciences, Taikang Center for Life and Medical Sciences, Taikang Medical School, Wuhan University, Wuhan, China

**Keywords:** LDL, lipids, cholesterol trafficking, lipid transfer proteins, Golgi apparatus, genome-wide CRISPR screen, lysosomal protein sorting, nucleotide sugar transporter

## Abstract

Accurate intracellular cholesterol traffic plays crucial roles. Niemann Pick type C (NPC) proteins NPC1 and NPC2, are two lysosomal cholesterol transporters that mediate the cholesterol exit from lysosomes. However, other proteins involved in this process remain poorly defined. Here, we find that the previously unannotated protein TMEM241 is required for cholesterol egressing from lysosomes through amphotericin B-based genome-wide CRISPR-Cas9 KO screening. Ablation of *TMEM241* caused impaired sorting of NPC2, a protein utilizes the mannose-6-phosphate (M6P) modification for lysosomal targeting, resulting in cholesterol accumulation in the lysosomes. TMEM241 is a member of solute transporters 35 nucleotide sugar transporters family and localizes on the *cis*-Golgi network. Our data indicate that TMEM241 transports UDP-N-acetylglucosamine (UDP-GlcNAc) into Golgi lumen and UDP-GlcNAc is used for the M6P modification of proteins including NPC2. Furthermore, *Tmem241*-deficient mice display cholesterol accumulation in pulmonary cells and behave pulmonary injury and hypokinesia. Taken together, we demonstrate that TMEM241 is a Golgi-localized UDP-GlcNAc transporter and loss of *TMEM241* causes cholesterol accumulation in lysosomes because of the impaired M6P-dependent lysosomal targeting of NPC2.

LDL-derived cholesterol must be released from lysosomes otherwise the lysosomal cholesterol accumulation causes diseases, such as Niemann-Pick disease type C (NPC) (1). Patients with NPC develop enlarged spleen and liver, progressive neurological symptoms, and premature death (2). NPC is dominantly caused by mutations in *NPC1* or *NPC2* genes. Human *NPC1* encodes a lysosomal transmembrane protein of 1,278 amino acids (AAs) and *NPC2* encodes a soluble protein of 151 AAs, which is concentrated in lysosomes and secreted from cells (3, 4, 5, 6).

Once delivered to lysosomes, the LDL particles are hydrolyzed and cholesterol is released. In the lysosomal lumen, the hydrocarbon tail of cholesterol first inserts deep into the binding pocket of NPC2. Further, cholesterol is handed over to the N-terminal domain binding pocket of NPC1 with its 3β-hydroxyl group buried. NPC1 employs a tunnel to deliver the cholesterol to insert into the lysosomal membrane (5, 7). Then cholesterol is transported to other organelles mainly by nonvesicular transport mechanism at membrane contact sites (8, 9, 10, 11, 12).

Adequate mannose-6-phosphate (M6P) modification is necessary for the lysosomal targeting of NPC2, otherwise NPC2 is secreted into medium (13). After synthesized in the endoplasmic reticulum (ER), NPC2 is delivered to the Golgi network, where it undergoes M6P modification. To support M6P modification, the UDP-N-acetylglucosamine (UDP-GlcNAc) is transported by its solute transporter 35A3 (SLC35A3) in the Golgi network (14), and GlcNAc-1-phosphotransferase (GNPT) selectively adds GlcNAc-1-phosphate (GlcNAc-1-P) to specific mannose residues of cargo proteins (15). Then GlcNAc is removed by the enzyme N-acetyglucosamine-1-phosphodiester α-N-acetylglucosaminidase. M6P monoesters are recognized by two M6P receptors (MPRs) in the *trans*-Golgi network, followed by lysosomal targeting (16, 17). About 5–20% of newly synthesized lysosomal proteins escape from MPRs binding in the Golgi and are secreted out of the cell (18).

In this study, we performed genome-wide CRISPR screening to seek for new cholesterol transport regulators under amphotericin B (AmB)-based selection. We found a function-unreported protein TMEM241 was involved in lysosomal transportation of cholesterol. *TMEM241* ablation led to lysosomal cholesterol accumulation and impaired lysosomal sorting of NPC2. We then demonstrated that TMEM241 was a Golgi-localized UDP-GlcNAc transport. Loss of *TMEM241* decreased M6P modification of many proteins, including NPC2. In summary, we identified TMEM241 contributes to lysosomal targeting of NPC2 by regulating the M6P modification pathway thus promotes lysosomal exit of LDL-derived cholesterol.

## Materials and methods

### Cell culture

HeLa or stably expressing lentiCas9-FLAG (HeLa/Cas9), SV589, and HEK293T cells were grown in a monolayer at 37°C in 5% CO_2_. The cells were maintained in medium A (DMEM containing 100 units/ml penicillin and 100 μg/ml streptomycin sulfate) supplemented with 10% FBS. HeLa/site-1 protease (*S1P)* KO cells were maintained as previously described (19). Cholesterol-depleting medium was medium A supplemented with 5% lipoprotein-deficient serum (to eliminate exogenous cholesterol absorption) supplemented with 1 mM lovastatin (to inhibit endogenous cholesterol biosynthesis) and 10 mM mevalonate (to permit the synthesis of nonsterol isoprenoids essential for cell growth) (20). For LDL and U18666A treatment, 50 μg/ml LDL and 1 μg/ml U18666A were added to the cholesterol-depleting medium for 4 h. Cyclodextrin medium was cholesterol-depleting medium supplemented with 1.5% hydroxypropyl-beta-cyclodextrin (HPCD) for 10 min (21). AmB medium was cholesterol-depleting medium supplemented with 300 μg/ml AmB.

### Antibodies

Anti-LAMP1 (H4A3-c) was from Developmental Studies Hybridoma Bank. Anti-NPC1 (13926-1-AP), anti-TOM20 (11802-1-AP) and anti-GAPDH (10494-1-AP), anti-cathepsin D (CTSD), and rabbit anti-FLAG (20543-1-AP) were from Proteintech Group. Anti-NPC2, mouse anti-FLAG (F9291) and anti-Actin (A1978) were from Sigma-Aldrich. Anti-GM130 (610,823) and anti-EEA1 (610,457) were from BD Transduction Laboratories. Anti-P230 was from BD Biosciences. Mouse anti-myc (sc-40) was from Santa Cruz. The myc-tagged single-chain M6P antibody fragment was obtained from Dr Thomas Braulke laboratory (University Medical Center Hamburg-Eppendorf, Germany) (18). And the monoclonal antibody against SREBP2 (1D2) were prepared in our laboratory (22). Horseradish peroxidase–conjugated donkey anti-mouse and anti-rabbit IgG were from Jackson Immuno Research Laboratories. These antibodies described above were used at dilutions of 1:500 for immunofluorescence staining and 1:1,000 for Western blot. Alexa Fluor 488 donkey anti-rabbit IgG and Alexa Fluor 555 donkey were from Life Technologies.

### Reagents

AmB, U18666A, crystal violet, lovastatin, sodium mevalonate, and filipin were from Sigma-Aldrich. 2-HPCD was from Cyclodextrin Technologies Development. Puromycin was from Biosharp. Human LDLs and lipoprotein-deficient serum (d > 1.215 g/ml) were prepared as previously described (23, 24). H&E staining kit (#C0105) was from Beyotime.

### Plasmids

LentiCas9-FLAG plasmid and human genome-scale CRISPR/Cas9 KO (GeCKO) pooled library (v2) (No. 1000000049) were from Addgene. The coding regions of *Tmem241* and *Slc35a3* were amplified from mouse liver cDNA using standard PCR and cloned into pHAGE-3×FLAG vectors. The plasmids plvx-NPC2-IRES-Zsgreen and pcDNA3-NPC1-EGFP were obtained as previously described (25).

### AmB selection procedure

HeLa/Cas9 stable cells were cultured in cholesterol-depleting medium for 16 h (it ensured depletion of cellular cholesterol and drastically induced the LDL receptor (LDLR) expression) and incubated with 30 μg/ml LDL and 1 μg/ml U18666A for 4 h at 37°C (large amounts of LDL-derived cholesterol were internalized by LDLR-mediated endocytosis and trapped in late endosome/lysosomes (LE/Lys) because of U18666A (26)). Then the cells were washed with PBS and treated with 1.5% HPCD for 10 min to acutely deplete cholesterol from plasma membrane (PM). Later, the cells were washed with PBS twice and maintained in cholesterol-depleting medium that U18666A was removed that allowed cholesterol in LE/Lys to transport to other organelles including PM. After 0 h, 1 h, 3 h, and 5 h, the cells were incubated with 300 μg/ml AmB medium for 45 min, which can specifically bind PM-cholesterol and form pores resulting in cytoplasmic leakage and cell death. The rates of cholesterol trafficking in cholesterol trafficking defective (CTD) cells were slower than in WT cells and their PM-cholesterol levels were lower than WT cells at certain time points. Thus, these CTD cells can survive from AmB treatment. Finally, the cells were washed with PBS twice and maintained in growth medium for 72 h. After five rounds of AmB selection, the surviving cells and the cells without selection were amplified and subjected to deep sequencing.

### Crystal violet staining

After exposed to 300 μg/ml AmB for 45 min, the cells were washed with PBS twice, then cultured in medium A supplemented with 10% FBS for 3 days. Survival cells were washed twice with PBS and fixed with 95% ethanol for 30 min at room temperature. Thereafter, cells were stained with 0.5% crystal violet in 25% ethanol for 1 h and washed with pure water three times, extracting dye with 10% acetic acid. The absorbance was determined at 600 nm.

### Genome-wide CRISPR/Cas9 screen and analysis

HeLa cells expressing Cas9 were infected with GeCKO v2.0 pooled libraries consist of over 122,411 single-guide RNA (sgRNA) constructs targeting more than 19,050 human genes at 0.3 multiplicity of infection. Infected cells were selected puromycin (4 μg/ml) for 4 days. After five rounds of AmB selections, survived populations were collected, genomic DNA was isolated from all samples, and the sgRNA sequences were amplified by PCR and sequenced on an Illumina NovaSeq 6000 and data were analyzed using the MAGeCK algorithm (27). As we intended to validate hit genes found in our screen, hit genes were selected as interesting candidates among those with a *P*-value <0.001 and log_2_ (fold change) >1 and at least two different enriched sgRNAs for genes enriched (see supplemental Table S1).

### RNA interference

Duplexes of siRNAs targeting human genes were synthesized by Ribobio (Guangzhou, China). All siRNA sequences are listed in supplemental Table S2. SV589 cells were plated on day 0 and transfected with siRNA oligonucleotides (50 nmol/ml) using RNAiMAX on day 1. After transfection for 48 h, the cells and media were harvested.

### Quantitative real-time PCR

Total RNA was extracted from cells or mouse tissues using Trizol (T9424, Sigma). The equal amounts of RNA from the same treatment were pooled for cDNA synthesis with oligo dT and reverse transcriptase MLV (Promega). Equal amounts of RNA were used for cDNA synthesis, followed by quantitative real-time PCR as previously described (28). The relative mRNA levels were calculated using the comparative cycle threshold method. Human *GAPDH* or mouse *18sRNA* were used as the control. All quantitative PCR primers are listed in supplemental Table S3. Gene expression was analyzed by quantitative real-time PCR on a Bio-Rad CFX384 Real-Time System.

### Immunofluorescence

Cells grown on glass coverslips were fixed with 4% paraformaldehyde (PFA) for 30 min at room temperature. For cellular NPC2 staining, cells were fixed with Bouin’s solution (Sigma) for 1 h. Then cells were washed with PBS, permeabilized with 0.1% Triton X-100 in PBS for 5 min, and blocked with 1% BSA in PBS for 1 h at room temperature. Cells were then incubated with primary antibodies for 1.5 h. After washing three times with PBS, cells were incubated with secondary antibodies or 1 h at room temperature. For filipin analysis, cells were incubated with 10% FBS/PBS containing 50 μg/ml filipin (block, cell membranes permeabilization, and labeling free cholesterol) after fixed with 4% PFA. And then cells were, respectively, labeled primary antibodies and fluorescent secondary antibodies in 10% FBS/PBS containing 50 μg/ml filipin for 1 h at room temperature. Last, cells were examined and imaged under a Leica Biosystems SP8 laser scanning microscope. The contours of cell were outlined manually, and background-subtracted fluorescent intensity was quantified using ImageJ.

### Immunoblotting

Cells were harvested and homogenized with RIPA buffer supplemented with protease inhibitors. After centrifuging at 13,400 *g* for 10 min, the supernatants were collected, and the protein concentrations were determined using the BCA kit (Thermo Fisher Scientific). The supernatants were mixed with the membrane protein solubilization buffer (62.5 mM Tris-HCl, pH 6.8; 15% SDS; 8 M urea; 10% glycerol; 100 mM DTT) plus the 4× loading at 37°C for 30 min. Proteins were resolved by SDS-PAGE and transferred to PVDF membrane. Blots were blocked with 5% BSA or in TBS plus 0.075% Tween and probed with primary antibodies overnight at 4°C. After TBS plus 0.075% Tween wash, blots were incubated with secondary antibodies for 1 h at room temperature. For NPC2 protein analysis in cell extracts and culture media, cells were cultured in medium without FBS for 12 h. Later, the media and cells were harvested separately. Proteins in the media were concentrated with centrifugal filters (10 kDa). Culture media containing equal amounts of proteins were subjected to immunoblotting. To analyze M6P modification, cells were grown in DMEM/low Glucose (SH30021-01, Cytiva) containing 100 units/ml penicillin and 100 μg/ml streptomycin sulfate without FBS for 12 h at 37°C before harvested.

### SREBP2 cleavage analysis

HeLa cells and HeLa/*TMEM241* KO cells were incubated in cholesterol-depleting medium for 16 h, followed by incubation in cholesterol-depleting medium supplemented with 50 μg/ml LDL for 5 h at 37°C. In the last 1 h, the cells were treated with N-acetyl-leucinal-leucinal-norleucinal at a final concentration of 25 μg/ml. Then the cells were harvested for analysis of SREBP2 cleavage (precursor of SREBP2 and nuclear form of SREBP2).

### KO cell lines establishment and validation

The *TMEM241* KO cell lines and *SLC35A3* KO cells were constructed by transient cotransfection of sgRNAs targeting the *TMEM241* gene and *SLC35A3* gene, respectively, in HeLa/Cas9 cell. The *TMEM241* and *SLC35A3* sgRNA sequences were as follows: 5′-gtagctgaagttatcatctg-3′; 5′-aacctaaaatacgtttccct-3′. Single cells with puromycin (4 μg/ml) selection were seeded in 96-well plates. After cell expansion, the gene KO DNA fragments of target loci were independently amplified by PCR with a primer pair (*TMEM241*-seqF: 5′-agccccagcgtcattttatct-3′; *TMEM241*-seqR: 5′-gatgcaaggcacccaggtta-3′; *SLC35A3*-seqF: 5′-ccttctccctctcggtgtttt-3′; *SLC35A3*-seqR: 5′-agtcactgctggactaatcaat-3′). The purified PCR products were sequenced and analyzed.

### Evolutionary relationships of taxa

The evolutionary history was inferred using the neighbor-joining method. The bootstrap consensus tree inferred from 1,000 replicate is taken to represent the evolutionary history of the taxa analyzed. Branches corresponding to partitions reproduced in less than 50% bootstrap replicates are collapsed. The evolutionary distances were computed using the Jones-Taylor-Thornton matrix–based method and are in the units of the number of amino acid substitutions per site. This analysis involved 32 AA sequences. All positions with less than 95% site coverage were eliminated, that is, fewer than 5% alignment gaps, missing data, and ambiguous bases were allowed at any position (partial deletion option). There were a total of 296 positions in the final dataset. Evolutionary analyses were conducted in MEGA11.

### Golgi isolation

Stable cell lines expressing TMEM241 or SLC35A3 by lentiviral particles and corresponding control cells were set up on day 0 at 37.5 × 10^5^ cells per 15 cm dish. On day 1, the cells were treated with DMEM/low Glucose (SH30021-01, HycloneTM) containing 100 units/ml penicillin and 100 μg/ml streptomycin sulfate without FBS for 12 h before harvested. The Golgi apparatus was isolated on day 2 as previously described with minor modifications (29). All operations were performed at 0–4°C. After treatment, cells were transferred into fresh ice-cold 0.5 M sucrose homogenization medium (prepared in buffer A: 37.5 mM Tris-maleate, pH 6.5, 1% dextran, 5 mM MgCl_2_, and 5 mM 2-mercaptoethanol) containing protease inhibitors for homogenizing. The homogenates were centrifuged at 1,000 *g* for 10 min to remove the nuclei and cellular debris. Then resulting homogenate supernatants were layered over 1.5 volumes of 1.25 M sucrose (prepared in buffer A) in Ultra clear tubes and were centrifuged at 100,000 *g* in a SW55i rotor for 60 min at 4°C. The crude Golgi apparatus fractions (F2) which appeared as a cloudy band at the 0.5 M sucrose (F1)/1.25 M sucrose (F3) interface were collected and diluted with 0.25 M sucrose homogenization medium and centrifuged at 25,000 *g* for 30 min to get pellets of crude Golgi sample.

### UDP-GlcNAc analysis by MS

The pellet of crude Golgi was washed with PBS, and then half was taken for measurement of protein concentration by BCA, and the other half was added with 80% methanol containing internal standard (IS) for detection of UDP-GlcNAc by LC-MS/MS. The mixture was vortexed for 30 s, sonicated for 10 min on ice, and repeated once. The samples were centrifuged at 12,000 rpm for 10 min at 4°C; and the supernatant was transferred into a clean tube to dryness using a speedvac (Labconco, USA). The dried samples were resuspended in 80% methanol and analyzed by ultraperformance liquid chromatography with MS/MS (UPLC–MS/MS) conducted on a Waters Acquity UPLC-system coupled with 5500 QTRAP system (SCIEX). Chromatographic separation was achieved on a Waters Acquity UPLC BEH C18 Column (2.1 mm × 100 mm, 1.7 μm, Waters) using a flow rate of 0.3 ml/min at 40°C; during an 8 min gradient (0–1 min 5% B, 1–5 min from 5% B to 50% B, 5–6 min from 50% B to 5% B, 6–8 min 5% B), using buffer A [LC-MS grade water with 0.3% formic acid (pH 9.0) with ammonium hydroxide] and buffer B (100% acetonitrile). MS was operated in negative ion mode using an ESI source. The parameters in the source were set as follows: curtain gas, 35 psi; collision gas, medium; ionspray voltage, −4,500 V; temperature, 500°C; ion source gas 1, 55 psi; ion source gas 2, 55 psi. The analytes were monitored in multiple reaction monitoring mode using the precursor-to-product ion transitions of *m/z* 606.0→384.9 for UDP-GlcNAc and *m/z* 118.9→73.8 for succinate-1,4–^13^C_2_ (IS). Collision energy was −38.29 eV for UDP-GlcNAc and −16.30 eV for IS. Peak determination and area integration were performed using Analyst 1.7.1 (SCIEX) and SCIEX OS 1.4.0 software (SCIEX). The protein and IS quantifications were used for normalization.

### Animals

All animal experiments were performed under the protocols approved by the Institutional Animal Care and Use Committee of Wuhan University.

*Tmem241* heterozygous (*Tmem241*^+/−^) mice on C57BL/6J background were generated by GemPharmatech (Nanjing China), using CRISPR/ Cas9–based technology. In brief, the sgRNAs targeting the sites flanking exon 6–exon 10 were coinjected with the Cas9 mRNA into zygotes, resulting in the depletion of exon 6–exon 10 and early stop of *Tmem241* in mice. Male and female Tmem241^+/−^ mice were crossed to generate *Tmem241 KO* (*Tmem241*^−/−^) mice and WT littermates. Mice were housed in a specific pathogen-free, temperature-controlled room with a 12-h light and 12-h dark-cycle. Mice were fed on a chow diet and housed in a pathogen-free animal facility in plastic cages at 22°C, with a daylight cycle from 6 a.m. to 6 p.m.

### Histology

Mouse tissues were fixed in 4% PFA, embedded in paraffin and cut into 5 μm sections using a microtome (Leica RM2235), or saturated with 30% sucrose in PBS at 4°C and embedded in OCT compound for 8 μm frozen section with a cryostat (Leica CM 3050S). For histological analysis, paraffin sections were deparaffinized and stained with H&E. The frozen sections were then processed for filipin staining.

### Open field test

A 25 × 25 cm^2^ field was equally divided into 3 × 3 squares with the middle square designated as the center. The animals were put on to the middle square of the entire field and allowed to explore freely for 10 min. The movement was recorded by an overhead camera and the total distance traveled and time spent in the center were analyzed using EthoVision XT 10 (Noldus, Leesburg, VA) to analyze the moving activities of different groups of mice.

### Rotarod test

The balance and motor coordination of mice were tested on a rotarod machine as previously described (25). Prior to the test, mice were placed on the rotarod instrument for 10 min daily for consecutive three days to train them. After training, mice were placed on the rotating rod, which rotates with a gradual increasing speed of 0–40 rpm. The experiment was ended if the animals slip down from the rotating rod.

### The treadmill fatigue test

The analyses were carried out using a six-lane motorized treadmill. Aged animals were subjected to be acclimatized and trained on the treadmill at low intensity for 30 min daily for consecutive 7 days. After that, mice were put on a treadmill at high magnitude and the test was stopped when the mouse remained in the fatigue zone for more than 10 s. The time to exhaustion was determined from the beginning of the test.

### The published single-cell sequencing data analysis

The *TMEM241* and *NPC2* expression analysis in different cell type of normal lung was obtained by the online tools from cellxgene (https://cellxgene.cziscience.com/gene-expression) based on combined published single-cell lung atlas (30, 31, 32, 33).

### Statistical analysis

The data in this study were presented as mean ± SD values and analyzed using appropriate statistical methods with SPSS Statistics 21 software. For groups that met the assumption of normal distribution, the differences between two groups were assessed using the Student’s *t* test. Comparisons among multiple groups were conducted using one-way ANOVA, followed by either the Bonferroni post hoc test (for data with homogeneity of variance) or the Tamhane T2 post hoc test (for data with heteroscedasticity). In cases where the data did not follow a normal distribution, nonparametric tests were employed. The figure legends include the specific statistical methods used and the corresponding *P*-values for each figure panel. A *P*-value of less than 0.05 was considered statistically significant.

## Results

### Genome-wide CRISPR screen for genes affecting intercellular cholesterol trafficking

**Fig. 1:**
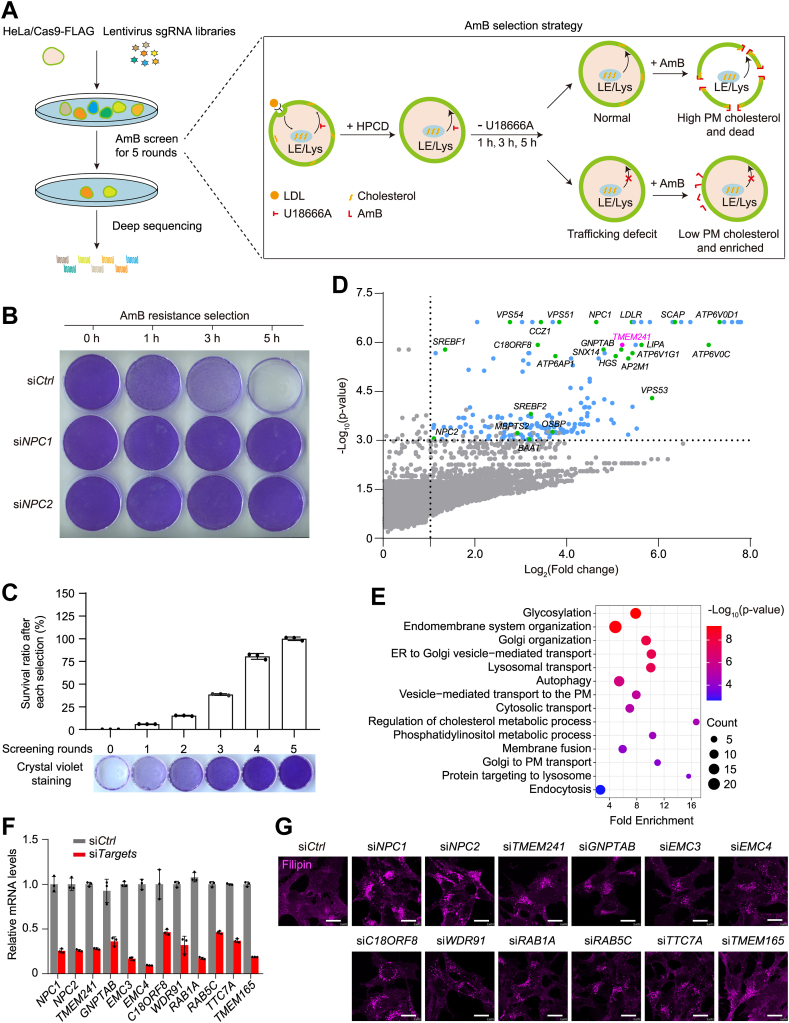
Genome-wide CRISPR-screen of factors in intracellular cholesterol trafficking. A: Schematic representation of the screen procedure. The HeLa/Cas9 cells were infected with lentiviruses expressing whole genome sgRNAs, followed by puromycin selection. The cell mixture was incubated in cholesterol-depleting medium overnight and then 30 μg/ml LDL containing 1 μM lovastatin and 1 μg/ml U18666A for 4 h. Then the cells were treated with 1.5% HPCD for 10 min and switched to cholesterol-depleting medium. After different time durations, the cells were treated with 300 μg/ml amphotericin B for 45 min. B: HeLa cells were transfected with control siRNA or siRNA targeting *NPC1* and *NPC2*, then subjected to the treatment in Fig. 1A. After removal of U18666A for 0, 1, 3, and 5 h, the cells were treated with amphotericin (B), washed, and stained with crystal violet. C: Survival ratio and crystal violet staining of the cells after every selection round. Results represent the mean ± SD of three independent experiments. D: Scatter plot showing for each enriched gene in (A). Genes with a phenotype value log_2_ (fold change) >1, *P*-value <0.001 and at least two different enriched sgRNAs are in blue, except for *TMEM241* in magenta and the known positive genes in green, the rest genes are in gray. The enriched genes were also listed in supplemental Table S1. E: Gene ontology (GO) term enrichment analysis for 179 genes with a phenotype value in (D). F: Q-PCR showing the knockdown efficiency of the representative candidates. G: The human fibroblast SV589 cells were transfected with indicated siRNAs for 48 h. The cells were fixed and stained with filipin. Scale bar, 10 μm. HPCD, hydroxypropyl-beta-cyclodextrin; sgRNA, single-guide RNA.

To uncover new regulators of cellular cholesterol transport, we adopted the GeCKO screening strategy combined with AmB selection (Fig. 1A). Briefly, the HeLa cell stably expressing Cas9-FLAG were infected with GeCKO v2.0 pooled libraries consisting of over 122,411 sgRNA constructs to target 19,050 human genes at a low multiplicity of infection (0.3) (34). Infected cells were subjected to the AmB-based screening that was developed before (10) (Fig. 1A). In brief, cells were incubated with cholesterol-depletion medium to inhibit endogenous cholesterol biogenesis and induce LDLR high expression. Second, a large amount of LDL-derived cholesterol was endocytosed and trapped in LE/Lys by addition of LDL and U18666A, a compound that reversibly blocks cholesterol efflux from LE/Lys. Next, the PM cholesterol was rapidly depleted by HPCD treatment, leading to synchronization of cells at the state of high cholesterol in LE/Lys and low cholesterol in PM. Last, cholesterol liberated from LDL exited LE/Lys and transported to the PM after removal of U18666A. Since AmB could bind to PM cholesterol, increase cell permeability, and cause cell death, WT cells were killed by AmB. But CTD cells were resistant to AmB as they had lower PM cholesterol.

To validate the screen strategy, HeLa cells received control siRNA or siRNA targeting *NPC1* and *NPC2* were subjected to the screen procedure. After removal of U18666A for different time periods, the cells were treated with AmB. Silencing *NPC1* or *NPC2* rendered complete AmB resistance as NPC1 and NPC2 are critical for cholesterol exiting lysosomes (8) (Fig. 1B).

After five rounds of selections, the survived cells were completely resistant to AmB treatment, and the sgRNA cassettes were amplified by PCR and subjected to deep sequencing (Fig. 1C). We filtered out 179 genes as top candidates with at least two independent sgRNAs and *P*-value <0.001, a log_2_ (fold change) of more than 1 (Fig. 1D and supplemental Table S1). Several reported genes involved in cholesterol trafficking and metabolism were highly enriched in our screening, such as: *NPC1* (lysosomal cholesterol transporter), *LDLR* (LDL endocytosis), *SREBP2* and *SCAP* (LDLR expression regulation), and *LIPA* (lysosomal cholesterol ester hydrolase). A previously uncharacterized gene *TMEM241* highlighted in magenta was also enriched. Gene ontology analysis indicated the enrichment of several pathways and the “glycosylation” and “Golgi organization” were identified among the top hits (Fig. 1E). We randomly selected 12 candidates, silenced them with siRNAs, and found drastic cholesterol accumulation in the cells compared with control siRNA (Fig. 1F, G).

### TMEM241 deficiency causes cholesterol transport defect

**Fig. 2:**
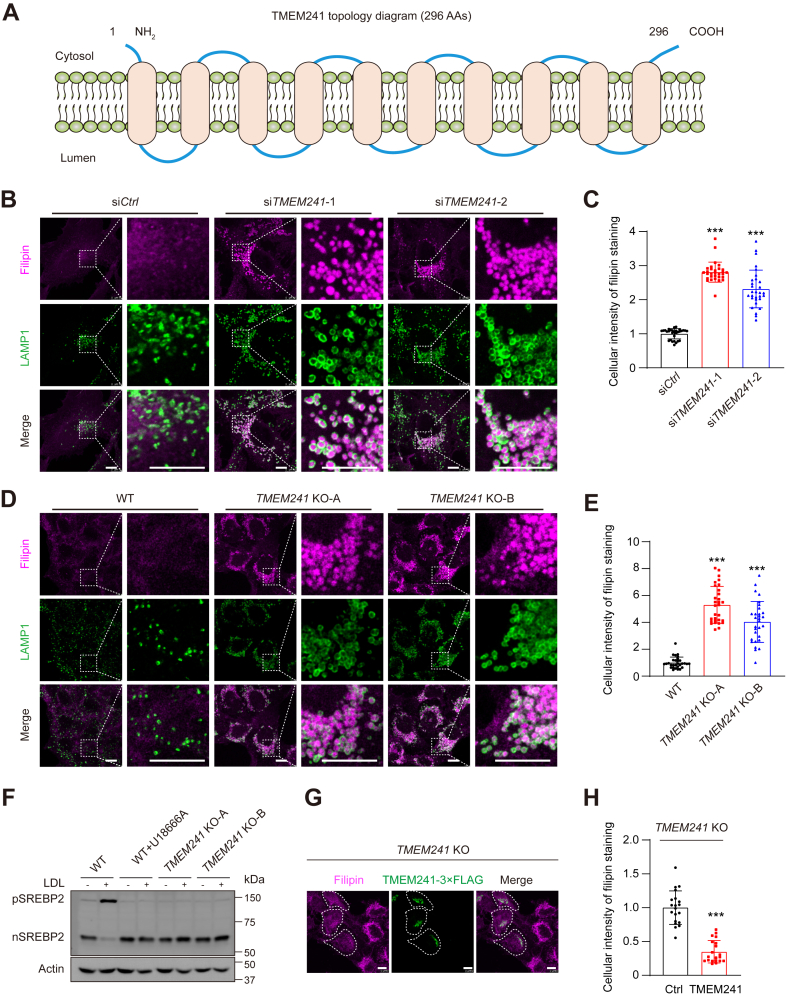
Deficiency of TMEM241 impaired lysosomal cholesterol transport. A: The predicted topology of TMEM241. B: SV589 cells were transfected with two independent *TMEM241* siRNAs for 48 h. The cells were fixed, stained with filipin (magenta), and immunostained with antibodies against endogenous LAMP1 (green). The enlarged images show at high magnification of the areas framed by a white dotted box. Scale bar, 10 μm. C: Quantification of the relative fluorescence intensity of filipin shown in (B). Data are normalized to control cells and represented as mean ± SD (n = 30). ∗∗∗*P* < 0.001, Student’s *t* test. D: HeLa cells and two lines of *TMEM241* KO cells generated by the CRISPR/Cas9 technique were stained with filipin (magenta) and immunostained with antibody against LAMP1 (green). The enlarged images show at high magnification of the areas framed by a white dotted box. Scale bar, 10 μm. E: Quantification of the relative fluorescence intensity of filipin shown in (D). Data are normalized to control cells and represented as mean ± SD (n = 30). ∗∗∗*P* < 0.001, Student’s *t* test. F: Analysis of SREBP2 cleavage. HeLa and HeLa/*TMEM241* KO cells were incubated in cholesterol-depleting medium for overnight and then treated with 50 μg/ml LDL for 5 h in the absence or presence of 1 μg/ml U18666A. In the last 1 h, 25 μg/ml ALLN was applied. G: HeLa/*TMEM241* KO cells were transfected with the plasmid expressing TMEM241-3×FLAG (green), fixed, and stained with filipin (magenta). The cells expressing TMEM241-3×FLAG is outlined. Scale bar, 10 μm. H: Quantification of the relative fluorescence intensity of filipin staining of the cells shown in (G). Data are normalized to control cells and represented as mean ± SD (n = 20). ∗∗∗*P* < 0.001, Student’s *t* test. ALLN, N-acetyl-leucinal-leucinal-norleucinal; nSREBP2, nuclear form of SREBP2; pSREBP2, precursor of SREBP2.

TMEM241 is a membrane protein consisting of ten putative transmembrane segments (Fig. 2A). Silencing *TMEM241* by siRNAs caused severe lysosomal cholesterol accumulation and pathological lysosome morphology (Fig. 2B, C and supplemental Fig. S1A). We also generated two *TMEM241* KO cell lines and they showed lysosomal cholesterol accumulation and swollen lysosomes compared with control cells (Fig. 2D, E and supplemental Fig. S1B). The *TMEM241* KO cells were also resistant to AmB (supplemental Fig. S1C). In WT cells, the LDL-derived cholesterol can leave lysosomes and transport to ER, where it caused ER retention of pSREBP2 and then decreased nuclear form of SREBP2. However, LDL failed to inhibit SREBP2 processing in *TMEM241* KO cells. As a positive control, U18666A blocked the lysosomal exit of cholesterol and ablated the inhibitory effect of LDL on SREBP2 maturation (Fig. 2F) (26). We transfected the plasmid expressing TMEM241 into *TMEM241* KO cells. Exogenous TMEM241 (green) successfully restored abnormal lysosomal cholesterol aggregation (Fig. 2G, H). Collectively, these data demonstrated that deficiency of *TMEM241* causes cholesterol accumulation in lysosomes, thereby impairing cholesterol transport to PM and ER.

### *TMEM241* deficiency decreases NPC2 in lysosomes

**Fig. 3:**
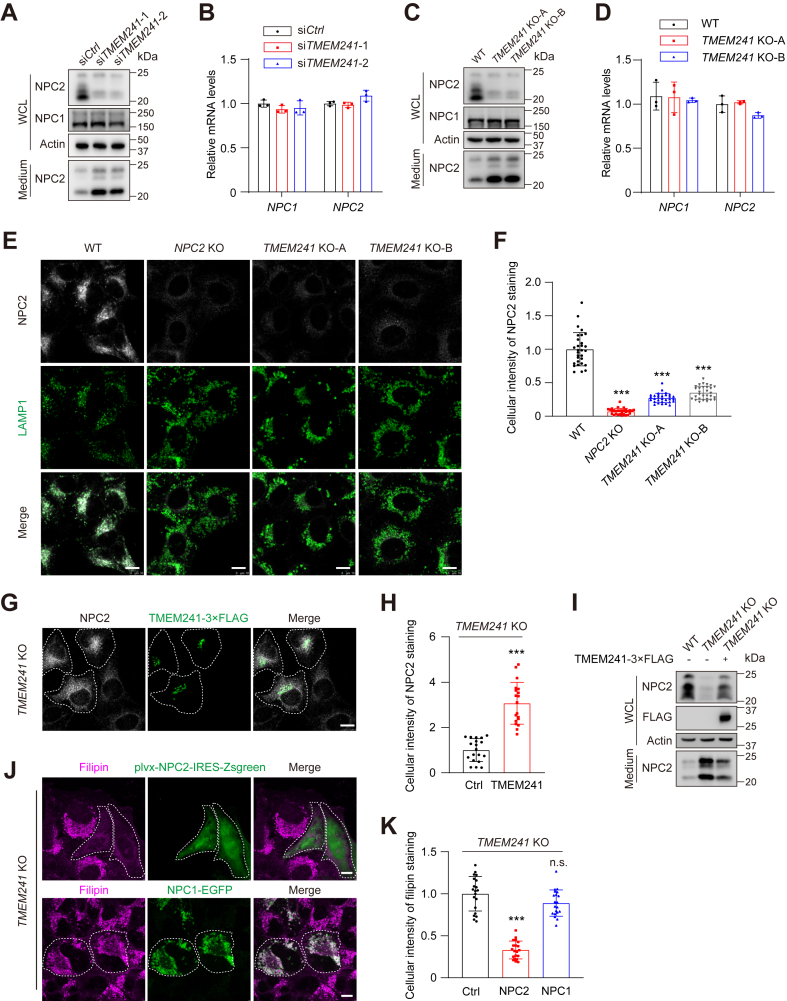
Deficiency of TMEM241 decreased lysosomal NPC2 and increased extracellular NPC2. A: The NPC1 and NPC2 proteins in the whole-cell lysate and culture medium of *TMEM241* knockdown cells and control cells. WCL, whole-cell lysate. B: Relative *NPC1* and *NPC2* mRNA levels in control and *TMEM241* knockdown cells. Data are normalized to control cells and represented as mean ± SD. C: The NPC1 and NPC2 proteins in the whole cell lysate and culture medium of HeLa/*TMEM241* KO cells and wild type cells. WCL, whole-cell lysate. D: Relative *NPC1* and *NPC2* mRNA levels in HeLa/*TMEM241* KO cells and WT cells. Data are normalized to control cells and represented as mean ± SD. E: HeLa/*NPC2* KO cells, HeLa/*TMEM241* KO cells, and WT cells were stained with endogenous anti-NPC2 (white) and anti-LAMP1 (green) antibodies. Scale bar, 10 μm. F: Quantification of the relative fluorescence intensity of NPC2 shown in (E). Data are normalized to control cells and represented as mean ± SD (n = 30). ∗∗∗*P* < 0.001, Student’s *t* test. G: HeLa/*TMEM241* KO cells were transfected with the plasmid expressing TMEM241-3×FLAG (green), fixed and stained with anti-NPC2 antibodies (white). The cells expressing TMEM241-3×FLAG is outlined. Scale bar, 10 μm. H: Quantification of the relative fluorescence intensity of NPC2 shown in (G). Data are normalized to control cells and represented as mean ± SD (n = 20). ∗∗∗*P* < 0.001, Student’s *t* test. I: The NPC2 protein in the whole-cell lysate and culture medium of WT cells, HeLa/*TMEM241* KO cells, and the *TMEM241* KO cells expressing TMEM241-3×FLAG. J: Filipin staining of HeLa/*TMEM241* KO cells transfected with the plasmid expressing NPC1 (green) or NPC2 (green). The cells expressing NPC1 or NPC2 are outlined. Scale bar, 10 μm. K: Quantification of the relative fluorescence intensity of filipin staining shown in (J). Data are normalized to control cells and represented as mean ± SD (n = 20). ∗∗∗*P* < 0.001, Student’s *t* test.

NPC1 and NPC2 are two key proteins required for lysosomal cholesterol exiting lysosomes. We then analyzed the expression of NPC1 and NPC2 in *TMEM241* knockdown cells. Without changing *NPC1* or *NPC2* mRNA levels, silencing *TMEM241* reduced the NPC2 protein level in whole-cell lysate but increased its level in culture medium. The NPC1 protein level in whole-cell lysate was not affected and not detected in medium (Fig. 3A, B). Similar results were observed in *TMEM241* KO cells (Fig. 3C, D). Immunofluorescence analysis showed that the level of NPC2 in lysosomes was reduced in *TMEM241* KO cells and the *NPC2* KO cells were used as the control group (Fig. 3E, F). Reexpression of TMEM241 in the *TMEM241* KO cells increased the lysosomal distribution of NPC2 and decreased extracellular NPC2 (Fig. 3G–I). Notably, overexpression of NPC2 but not NPC1 reversed the abnormal cholesterol accumulation in *TMEM241* KO cells (Fig. 3J, K). Thus, these results suggested that *TMEM241* deficiency causes lysosomal cholesterol accumulation through decreasing the lysosomal targeting of NPC2.

### TMEM241 is a Golgi-localized UDP-GlcNAc transporter

**Fig. 4:**
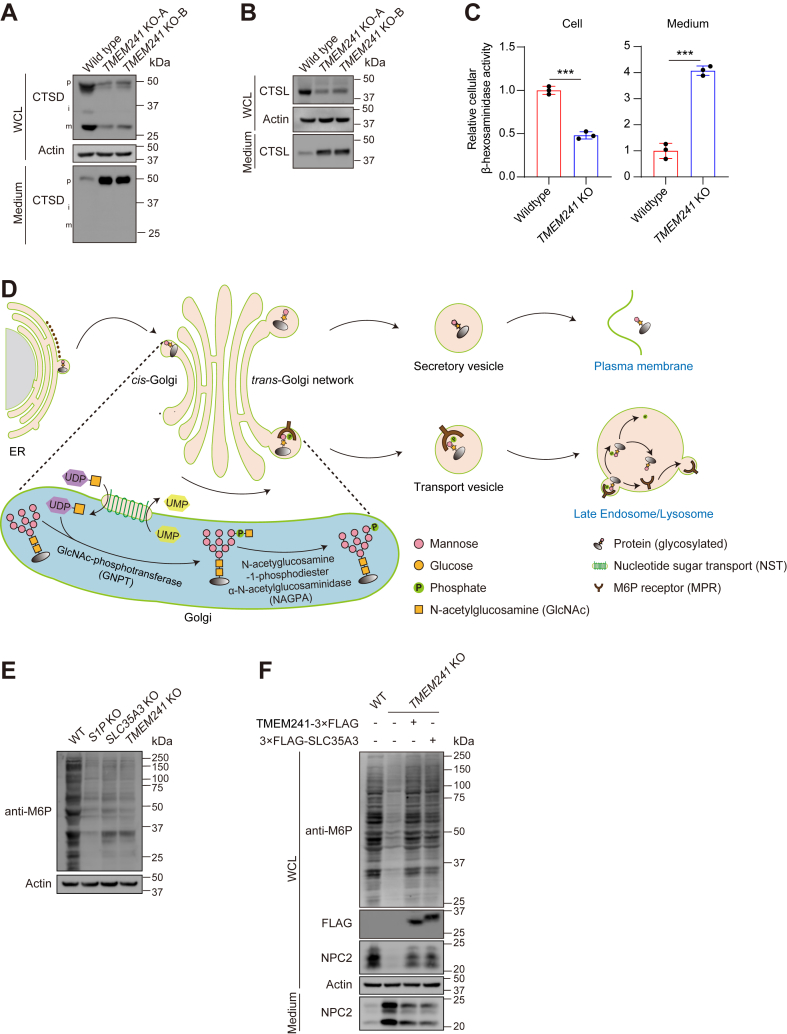
TMEM241 performs as an UDP-GlcNAc transporter to regulate NPC2 lysosomal targeting. A, B: Western blot analysis of lysosomal hydrolases: cathepsin D (CTSD) and cathepsin L (CTSL) in the whole-cell lysate and culture medium of HeLa/*TMEM241* KO cells and WT cells. On the left, different forms of Cathepsin D are denoted as follows: “p” for precursor, “i” for intermediate, and “m” for mature heavy chain. WCL, whole-cell lysate. C: The relative activity of lysosomal enzyme β-hexosaminidase in whole-cell lysates and culture media of control and *TMEM241* KO cells. Results are normalized to the protein amounts. Data are represented as mean ± SD (n = 3). ∗∗∗*P* < 0.001, Student’s *t* test. D: Diagrammatic sketch of M6P-dependent lysosomal targeting of the luminal proteins. E: Immunoblot analysis of HeLa, HeLa/*S1P* KO, HeLa/*SLC35A3* KO, and HeLa/*TMEM241* KO cells using the anti-M6P antibody and actin antibody. F: Immunoblot analysis of WT cells and the *TMEM241* KO cells stably expressing TMEM241-3×FLAG or 3×FLAG-SLC35A3. M6P, mannose-6-phosphate; NPC, Niemann-Pick disease type C; UDP-GlcNAc, UDP-N-acetylglucosamine; WCL, whole-cell lysate.

Besides NPC2, the lysosomal proteins, CTSD and cathepsin L (CTSL), showed reduced levels in cells and increased levels in medium. Similarly, the enzyme activity of lysosomal β-hexosaminidase in whole-cell lysate was decreased but increased in medium (Fig. 4A–C).

M6P posttranslational modification is required for trafficking soluble proteins into lysosomes. The M6P modification occurs in a two-step pathway in the lumen of Golgi. First, GlcNAc-1-P is transferred from UDP-GlcNAc to the 6-position of mannose residues of N-linked oligosaccharides by GlcNAc-phosphotransferase. Second, the terminal GlcNAc is removed by the uncovering enzyme N-acetyglucosamine-1-phosphodiester α-N-acetylglucosaminidase (17). The exposed M6P is recognized by the MPRs in *trans*-Golgi apparatus and delivered to lysosomes. UDP-GlcNAc is synthesized in cytosol and its translocation into Golgi lumen is a prerequisite for M6P modification. Previous studies have shown that nucleotide sugar transporters are encoded by the SLC35 gene family and SLC35A3 transports UDP-GlcNAc into the lumen of *cis*-Golgi network (35) (Fig. 4D; supplemental Figs. S2 and S3).

Evolutionary tree analysis suggested that TMEM241 belonged to SLC35 transporters family and displayed high relevance to SLC35A3 (supplemental Fig. S3). Therefore, we hypothesized that TMEM241 affected NPC2 location by acting as an UDP-GlcNAc transporter. We measured the gross M6P modification of cellular proteins with anti-M6P antibodies. The protease S1P cuts the α/β-subunit precursor of GlcNAc-phosphotransferase to generate mature GlcNAc-phosphotransferase and SLC35A3 transports UDP-GlcNAc into the Golgi lumen (19, 36, 37). So, the gross M6P was decreased in the *S1P* KO or *SLC35A3* KO cells. Surprisingly, the M6P modification of total cellular proteins was also reduced in the *TMEM241* KO cells (Fig. 4E). Reexpression of exogenous TMEM241 or SLC35A3 substantially increased the gross M6P modification and the cellular NPC2 level in *TMEM241* KO cells (Fig. 4F).

**Fig. 5:**
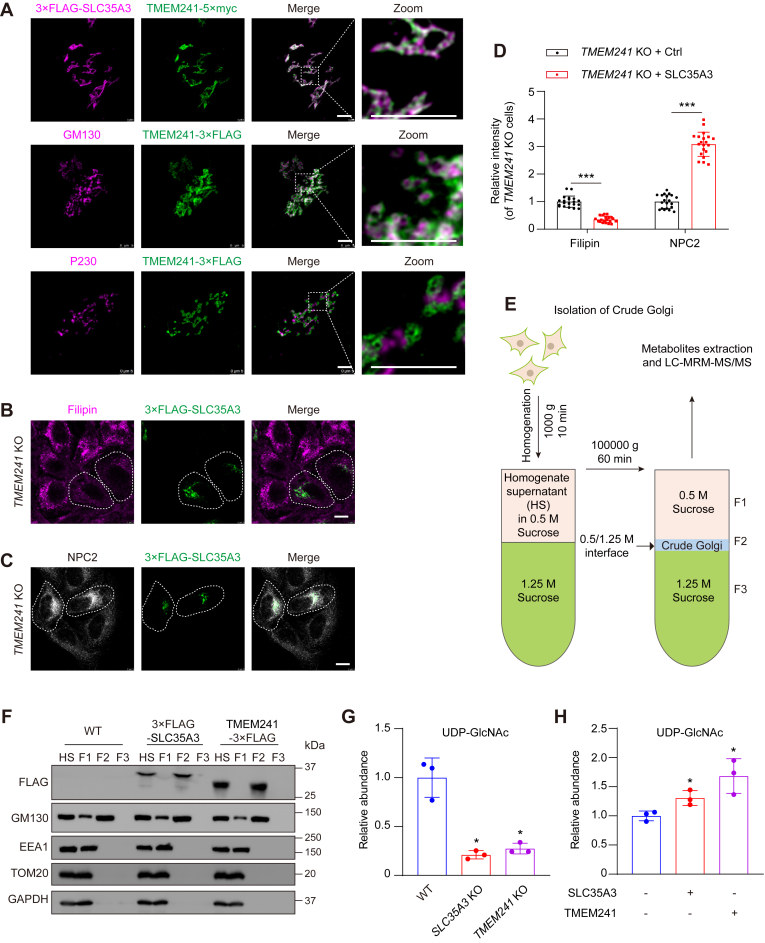
TMEM241 is a Golgi-localized UDP-GlcNAc transporter. A: SV589 cells were cotransfected with the plasmid encoding TMEM241-5×myc (green) and 3×FLAG-SLC35A3 (magenta) for 48 h, and immunostained with the antibody against myc and FLAG. SV589 cells were transfected with the plasmid encoding TMEM241-3×FLAG (green) for 48 h, and immunostained with the antibody against GM130 (*cis*-Golgi marker, magenta) and P230 (*trans*-Golgi network marker, magenta). The enlarged images show at high magnification of the areas framed by a white dotted box. Scale bar, 5 μm. B: *TMEM241* KO cells were transfected with 3×FLAG-SLC35A3 plasmid, fixed, and stained with anti-FLAG (green) and filipin (magenta). Scale bar, 10 μm. C: *TMEM241* KO cells were transfected with 3×FLAG-SLC35A3 plasmid, fixed, and stained with anti-NPC2 (white) and anti-FLAG (green). Scale bar, 10 μm. D: Relative intensity quantification of cellular filipin shown in (B) and cellular NPC2 shown in (C). Data are normalized to control cells and represented as mean ± SD (n = 20). ∗∗∗*P* < 0.001, Student’s *t* test. Scale bar, 10 μm. E: Schematic diagram depicting the strategy for detecting metabolites in crude Golgi isolated by sucrose density gradient centrifugation. F: Immunoblot analysis of different organelles level in homogenate supernatant (HS) and 0.5 M sucrose fraction (F1), the crude Golgi apparatus fraction (F2) and 1.25 M sucrose fraction (F3) of WT cells, TMEM241-overexpressing cells, and SLC35A3-overexpressing cells. G: The relative abundance of UDP-GlcNAc detected by LC-multiple reaction monitoring–MS/MS in crude Golgi isolated from control cells (blue), HeLa*/SLC35A3* KO cells (red) and HeLa/*TMEM241* KO cells (purple). Data are normalized to control cells and represented as mean ± SD (n = 3). ∗ for 0.01 ≤ *P* < 0.05, Student’s *t* test. H: The relative abundance of the UDP-GlcNAc detected by LC-MRM-MS/MS in crude Golgi isolated from *SLC35A3* KO cells (blue) and *SLC35A3* KO cells overexpressing SLC35A3 (red) or TMEM241 (purple). Data are normalized to control cells and represented as mean ± SD (n = 3). ∗ for 0.01 ≤ *P* < 0.05, Student’s *t* test. EEA1, endosome marker; GAPDH, cytosol marker; GM130, Golgi marker; NPC, Niemann-Pick disease type C; SLC35, solute transporter 35; TOM20, mitochondria marker; UDP-GlcNAc, UDP-N-acetylglucosamine.

Immunofluorescent analysis showed that TMEM241 colocalized with SLC35A3 and GM130, the *cis*-Golgi marker, but not with P230, the *trans*-Golgi network marker (Fig. 5A). The M6P reaction happens in the *cis*-Golgi network (17). Immunofluorescence detection proved that SLC35A3 can rescue intracellular cholesterol accumulation and NPC2 sorting abnormalities caused by *TMEM241* KO (Fig. 5B–D). Since *TMEM241* deficiency compromised lysosomal targeting of several luminal proteins due to impaired gross M6P which could be complemented by SLC35A3, it is likely that TMEM241 is a Golgi-localized UDP-GlcNAc transporter. To demonstrate this notion, we purified Golgi apparatus as illustrated in Fig. 5E. The F2 fraction contained Golgi with neglectable endosomes, mitochondria, or cytosol (Fig. 5F). LC-multiple reaction monitoring–MS/MS was used to analyze UDP-GlcNAc (supplemental Fig. S4A, B). The results showed that the UDP-GlcNAc in the Golgi from *SLC35A3* KO or *TMEM241* KO cells was decreased by 70–80% compared with WT cells (Fig. 5G and supplemental Fig. S4C). On the contrary, overexpression of SLC35A3 in *SLC35A3* KO cells increased Golgi UDP-GlcNAc by 30% and overexpression of TMEM241 increased UDP-GlcNAc in Golgi by 70% (Fig. 5H and supplemental Fig. S4D). These results demonstrated that TMEM241 is a Golgi-localized UDP-GlcNAc transporter.

### TMEM241 absence induced cholesterol accumulation and pulmonary injury

**Fig. 6:**
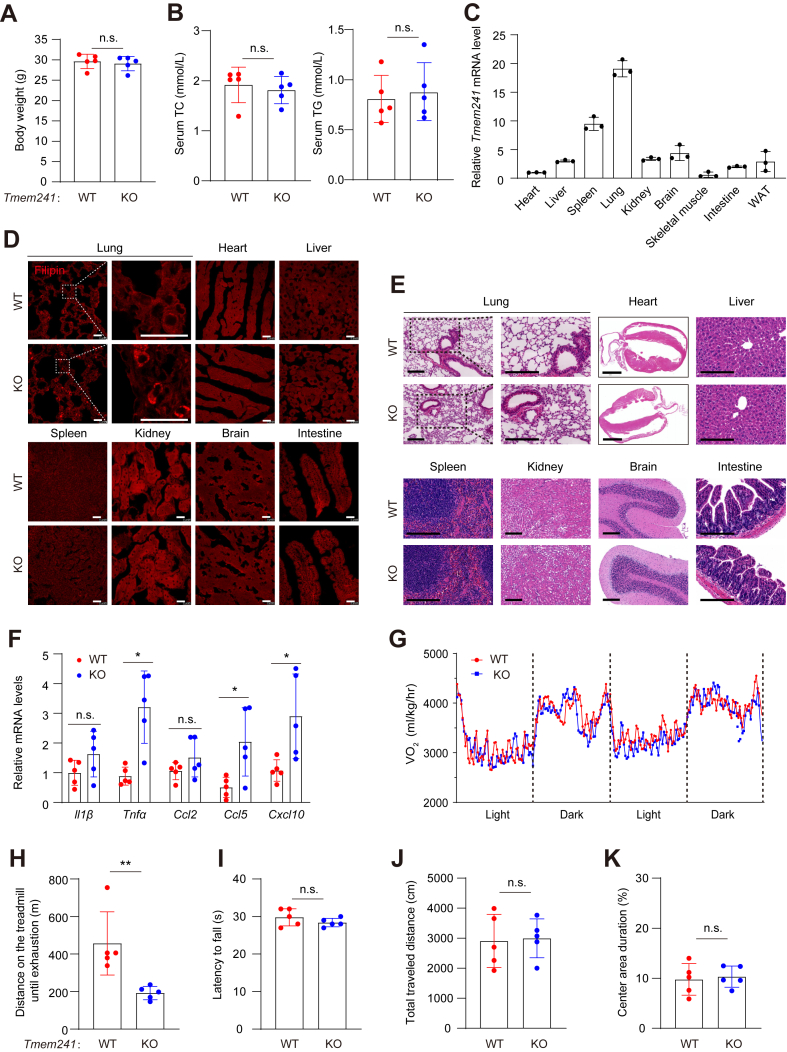
TMEM241 deficiency caused cholesterol accumulation in lung cells and lung inflammatory infiltration. A: Body weight under normal chow diet feeding (male, 8 months, n = 5 per group). n.s. = no significance. B: Serum total cholesterol (TC) and total triglyceride (TG) statistics of male WT and *Tmem241* KO mice at the age of 3 months (n = 5 per group). n.s. = no significance. C: Relative *Tmem241* mRNA levels in different organs and tissues. Data were normalized to heart tissue and presented as mean ± SD (n = 3). D: Filipin staining of different tissues of male WT and *Tmem241* KO mice at the age of 8 months. Scale bar, 25 μm. E: H&E staining of different tissues of male WT and *Tmem241* KO mice at the age of 8 months. Scale bar of heart, 2 mm; Scale bar of other tissues, 200 μm. F: Quantitative real-time PCR analysis of genes in the lungs of WT and *Tmem241* KO mice at the age of 3 months. Data were normalized to *IL1β* of WT mice and presented as mean ± SD (n = 5 independent trials). G: Metabolic cage analysis of male WT and *Tmem241* KO mice at quiescent state (n = 5 per group) at the age of 8 months n.s. = no significance. H: Treadmill test analysis of male WT and *Tmem241* KO mice (n = 5 per group) at the age of 8 months. ∗∗ for 0.001 ≤ *P* < 0.01, Student’s *t* test. I: Rotarod test analysis of WT and *Tmem241* KO mice at 8 months (n = 5 per group). n.s. = no significance. J, K: The total distance and center area duration traveled within the open field arena (25 × 25 cm^2^) was assessed over 10 min (n = 5 per group, at the age of 7 months). n.s. = no significance.

To investigate the function of TMEM241 in vivo, we generated whole body *Tmem241* KO mice by zygote injection with sgRNA and Cas9-coding mRNA. The mice were grossly normal and showed similar body weight, total cholesterol in serum, and triglyceride in serum with WT mice (Fig. 6A, B). The tissue expression profile showed that the lung had the highest *Tmem241* expression level (Fig. 6C). Intracellular cholesterol accumulation was detected in lung, not other tissues including the heart, liver, spleen, kidney, or brain in the *Tmem241*-deficient mice. Notably, cholesterol accumulation occurred in minor cells in lung (Fig. 6D). Accordingly, we did not detect any change in NPC2 level in the lung tissue and plasma (supplemental Fig. S5A). We speculated that TMEM241 only affects NPC2 in specific lung cells. Analysis of publicly available single-cell sequencing results of normal lung provided clues, indicating that *TMEM241* and *NPC2* were highly coexpressed in specific cell types, such as alveolar macrophage or stromal cells, rather than type I pneumocyte, type II pneumocyte, or club cells (supplemental Fig. S5B) (30, 31, 32, 33). The lung of *Tmem241*-deficient mouse showed inflammatory cell infiltration and thicker alveolar walls (Fig. 6E) that may impair respiratory function and cause hypokinesia. The expression of proinflammatory genes such as *TNFα*, *CCL5,* and *CXCL10* was increased in *Tmem241*-ablated mice (Fig. 6F). Although the metabolic levels in quiescent condition showed no significant differences in WT and *Tmem241*-deficient mice (Fig. 6G), the *Tmem241*-deficient mice showed less fatigue distance in treadmill test compared with WT mice (Fig. 6H). The functions of central nervous system seemed no significant differences compared with the control mice by rotarod test and open filed test (Fig. 6I–K). Collectively, *Tmem241* ablation caused ectopic cholesterol accumulation in lung cells. The inflammation was increased, and lung function might be impaired.

## Discussion

In this study, we performed CRISPR-based whole genome screening under AmB-based selection to seek for new cholesterol transport regulators. Analysis of sgRNA sequencing profiles displayed the enrichment of the canonical cholesterol trafficking regulators such as *LDLR*, *SCAP*, *SREBF1*, *SREBF2,* and *NPC1*. Many other reported regulators were also appeared in the top hits: the adaptor protein 2 complex (38), the ATPase complex (39) and GARP complex (*VPS51*, *VPS52*, *VPS53*, *VPS54*) (25), *AAGAB* that is involved in clathrin-coated vesicle trafficking (38), *Rab7*, *CCZ1B,* and *C18ORF8* (Fig. 1D) (40). Notably, *GNPTAB*, the gene encodes the key subunits of GNPT in the M6P modification pathway, was robustly enriched, indicating that the M6P modification pathway was closely associated with lysosomal cholesterol exit (Fig. 1D, F, G). These positive genes confirm the reliability of our screening.

Here, we identified TMEM241 regulates cholesterol trafficking. *TMEM241* ablation caused lysosomal cholesterol accumulation. We found that TMEM241 functioned as a UDP-GlcNAc transporter and a core component in M6P pathway. *TMEM241* ablation led to improper lysosomal targeting of NPC2. SLC35A3 is a well-known UDP-GlcNAc transporter (14). The absence of SLC35A3 resulted in cholesterol accumulation in lysosomes (supplemental Fig. S2A–D), which has not previously reported. Evolutionary tree analysis showed that TMEM241 was highly homologous with SLC35A3 (supplemental Fig. S4), and immunofluorescence assay indicated that both TMEM241 and SLC35A3 located at *cis*-Golgi (Fig. 5A). Significantly lowered M6P modification level in *TMEM241* KO cells demonstrated that TMEM241 is a crucial regulator for M6P modification. The SLC35A3 and TMEM241 restoring experiments together with MS quantification of UDP-GlcNAc in purified Golgi from cells under different SLC35A3 and TMEM241 expression levels strongly supported that TMEM241 is a new UDP-GlcNAc transporter. *TMEM241* was highly expressed in lung tissues. *Tmem241*-deficient mice behaved as abnormal cholesterol accumulation, lung inflammatory infiltration, and hypokinesia, suggesting the importance of lysosome homeostasis in lung function.

The deficiency of cation-dependent MPR and the cation-independent MPR were proved to cause NPC2 excessive secretion and lysosomal cholesterol accumulation (25, 41). The loss of the GARP complex disrupts the retrieval of cation-independent MPR to the Golgi, resulting in abnormal lysosomal sorting of NPC2 and impaired cholesterol transportation (25). Furthermore, GNPTAB impairment leads to abnormal intracellular cholesterol accumulation, as a pivotal enzyme in the formation of M6P modifications (Fig. 1F, G). The destruction of other factors responsible for GNPTAB maturation also resulted in abnormal cholesterol accumulation within lysosomes, such as S1P and POST1 (19, 42). These findings together with our findings here support the important role of M6P modification pathway in regulating lysosomal cholesterol exit.

We found that the food intake, body weight, serum TC, and triglyceride in *Tmem241* KO mice were negligibly changed. Rotarod test and open filed test suggested that central nervous system remained normal in *Tmem241* KO mice. However, NPC patients usually behave as neurological disorders and abnormal serum lipid profiles (1), indicating that TMEM241 is not a neurological regulator. We observed the highest *Tmem241* expression in the lung where abnormal cholesterol accumulation, inflammatory infiltration, and alveolar walls thickening occurred (Fig. 6C–F). However, we did not detect any change in NPC2 level in the lung tissue and plasma (supplemental Fig. S5A). The filipin staining results showed that cholesterol aberrantly accumulated only in a specific cell type in the lungs of *Tmem241* KO mice, while most lung tissue cells were indistinguishable (Fig. 6D). To clarify this issue, we adopted single-cell RNA-seq analysis. *TMEM241* expression is cell type–specific and rare in lung. The proportion of cells expressing TMEM241 is less than 2.59%. These data suggested that TMEM241 may be selectively expressed in a specific cell type within the lung, masking the differentiation of NPC2 as detected by Western blot analysis of the entire lung.

The lysosomal luminal proteins require M6P sorting pathway for lysosomal targeting (43). M6P pathway impairment causes unusual lysosomal storage disorder (44). As an indispensable organelle in which antigens from dead cells are cleaved and eliminated, lysosome homeostasis is necessary for autoimmune response (45). It has been well established that lysosomal enzymes are implicated in promoting clearance of dead cells and the production of cytokine (46). In cultured cells, we found reduced α-galactosidase activity and imbalanced CTSD /cathepsin L distribution in *TMEM241*-deficent group compared with the controls (Fig. 4A–C), which has been demonstrated to induce autoimmune response, such as aberrant activation of NKT cells by blunted degradation and excessive accumulation of antigens (47). We therefore conjectured that the inflammatory infiltration in the lung of *Tmem241* KO mice was owned to lysosome dysfunction.

In summary, this study shows that TMEM241 is a Golgi-localized UDP-GlcNAc transporter. Loss of *TMEM241* impairs M6P modification of proteins including NPC2. Therefore, the lysosomal targeting of NPC2 is decreased and cholesterol exits from lysosomes are blocked.

## Data availability

Raw deep-sequencing data have been deposited in the National Center for Biotechnology Information Sequence Read Archive (PRJNA1015588). Other data are included in this article and Supplemental information.

## Supplemental data

This article contains supplemental data.

## Conflict of interest

The authors declare that they have no conflicts of interest with the contents of this article.
