# Supplementary material for: TMEM241 is a UDP-N-acetylglucosamine transporter required for M6P modification of NPC2 and cholesterol transport
Source: J Lipid Res. 2023 Oct 27;64(12):100465. doi: 10.1016/j.jlr.2023.100465 (PMC10689955; doi:10.1016/j.jlr.2023.100465)
Supplement: Supplemental Figures S1–S5 and Tables S2 and S3 [file mmc1.docx]

**SUPPLEMENTAL INFORMATION:**

**TMEM241 is a UDP-N-acetylglucosamine transporter required for M6P modification of NPC2 and cholesterol transport**

Nan Zhao^1^, Gang Deng^1^, Pei-Xin Yuan^1^, Ya-Fen Zhang^1^, Lu-Yi Jiang^1^, Xiaolu Zhao^1, *^, Bao-Liang Song^1, *^

^1^ The Institute for Advanced Studies, Hubei Key Laboratory of Cell Homeostasis, College of Life Sciences, Taikang Center for Life and Medical Sciences, Taikang Medical School, Wuhan University, Wuhan 430072, China

*: Correspondence to: blsong@whu.edu.cn (B.-L.S.) and zhaoxiaolu@whu.edu.cn (X. L.Z.)

Supplementary Figures


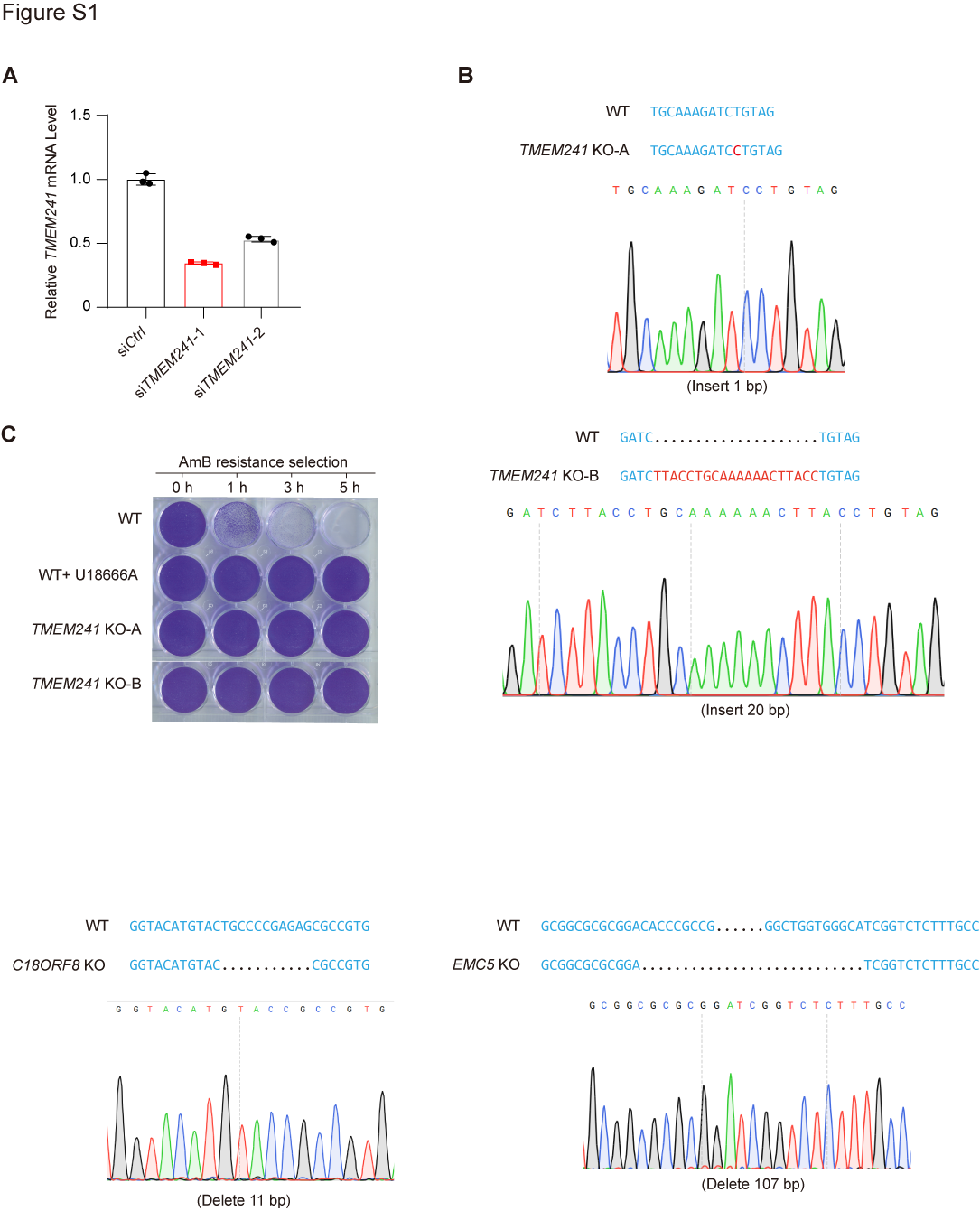


**Figure. S1 Validation of *TMEM241* knockdown efficiency, knockout cells and AmB selection.**

1. Quantitative real time PCR showing the knockdown efficiency of two *TMEM241* siRNAs. SV589 cells were transfected with scrambled siRNA for 48 h and harvested for Q-PCR analysis. The relative mRNA level of *TMEM241* was normalized to control that of *GAPDH* and presented as mean ± SD (n = 3).
2. DNA sequencing peaking maps of *TMEM241* knockout cell lines generated by the CRISPR/Cas9 technique in HeLa cells.
3. HeLa and HeLa/*TMEM241* KO cells were treated as shown in Figure 1A. After indicated time points, the cells were stained with crystal violet. In the second group, U18666A was not removed.

**
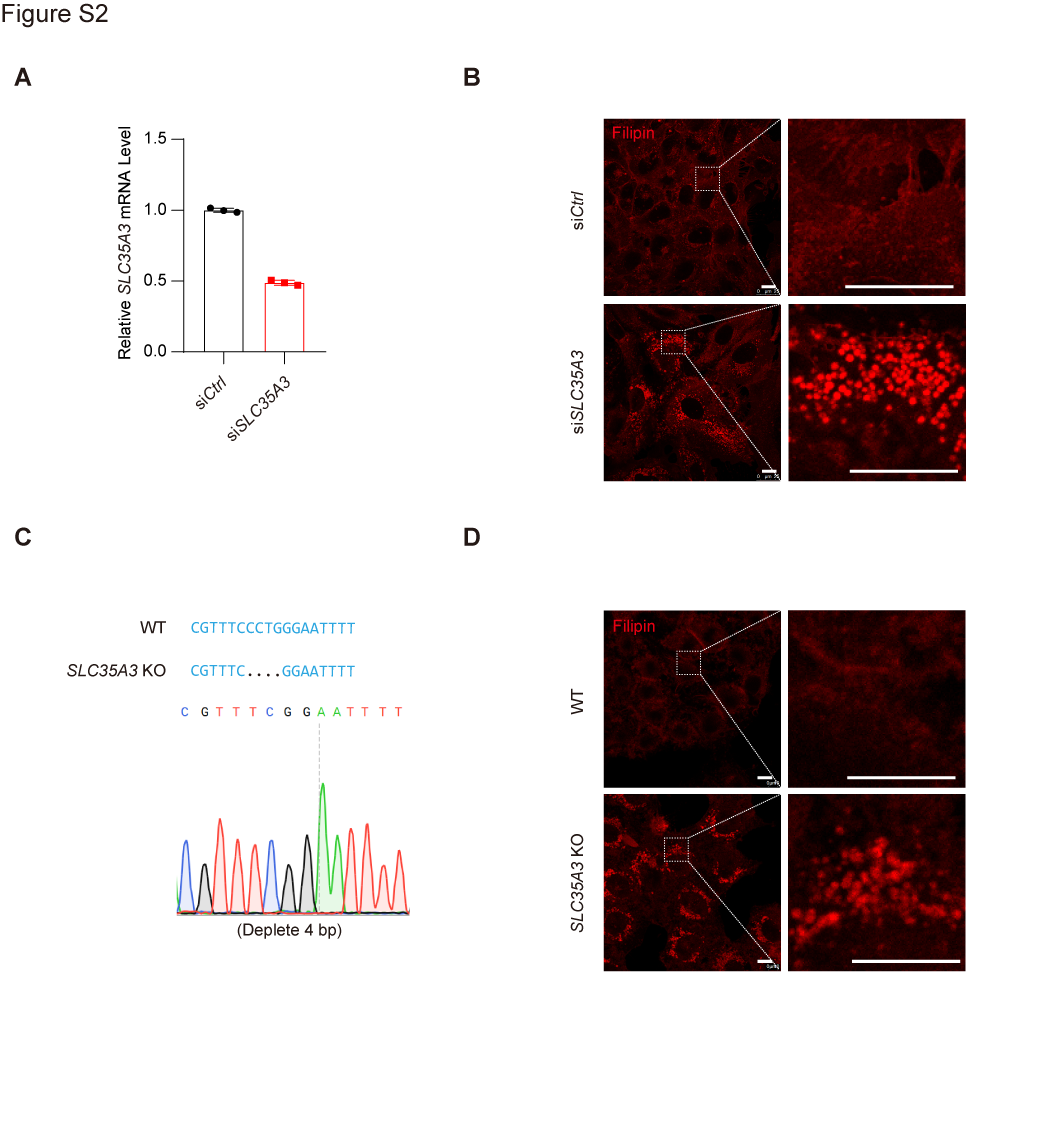
Figure. S2 *SLC35A3* deficiency causes abnormal cholesterol accumulation.**

1. Quantitative real time PCR showing the knockdown efficiency of *SLC35A3* siRNA. SV589 cells were transfected with scrambled siRNA for 48 h and harvested for Q-PCR analysis. The relative mRNA level of *SLC35A3* was normalized to control that of *GAPDH* and presented as mean ± SD (n = 3).
2. The human fibroblast SV589 cells were transfected with indicated siRNAs for 48 h. The cells were fixed and stained with filipin. Scale bar, 10 μm.
3. DNA sequencing peaking map of *SLC35A3* knockout cell line generated by the CRISPR/Cas9 technique in HeLa cells.
4. HeLa cells and *SLC35A3* KO cells were stained with filipin (red). The enlarged images show at high magnification of the areas framed by a white dotted box. Scale bar, 10 μm.


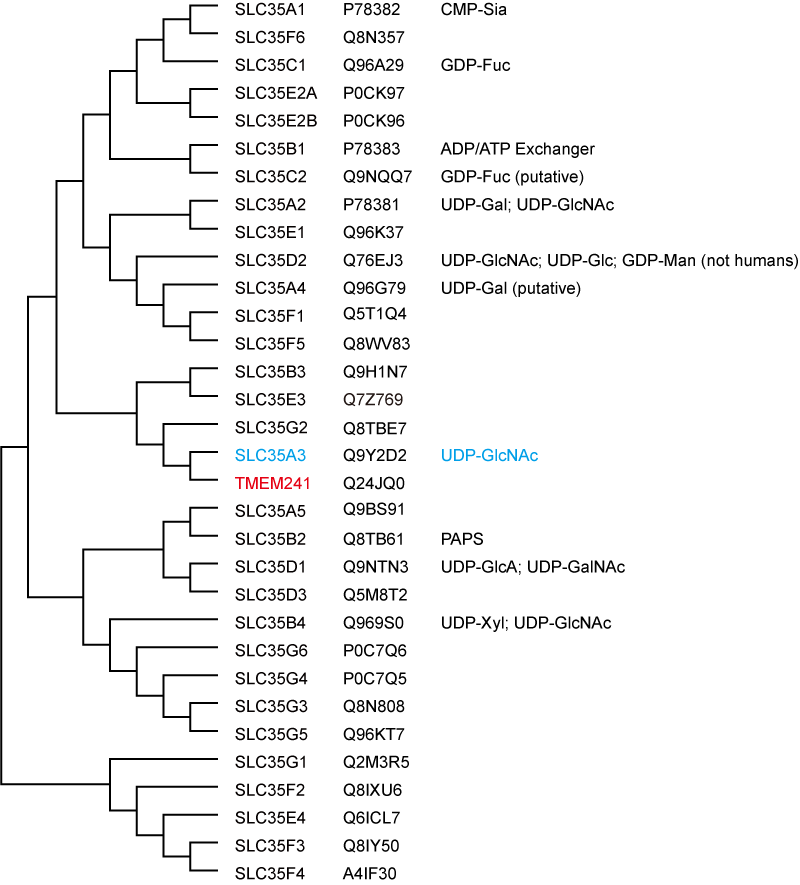


**Figure. S3 Evolutionary tree analysis.**

The sequences of TMEM241 and canonical nucleotide sugar transporters of SLC35 nucleotide sugar transporters family were subjected to conduct evolutionary tree analysis using the Neighbor-Joining method by MEGA11. The annotation number of all proteins and known substrates are shown.

**
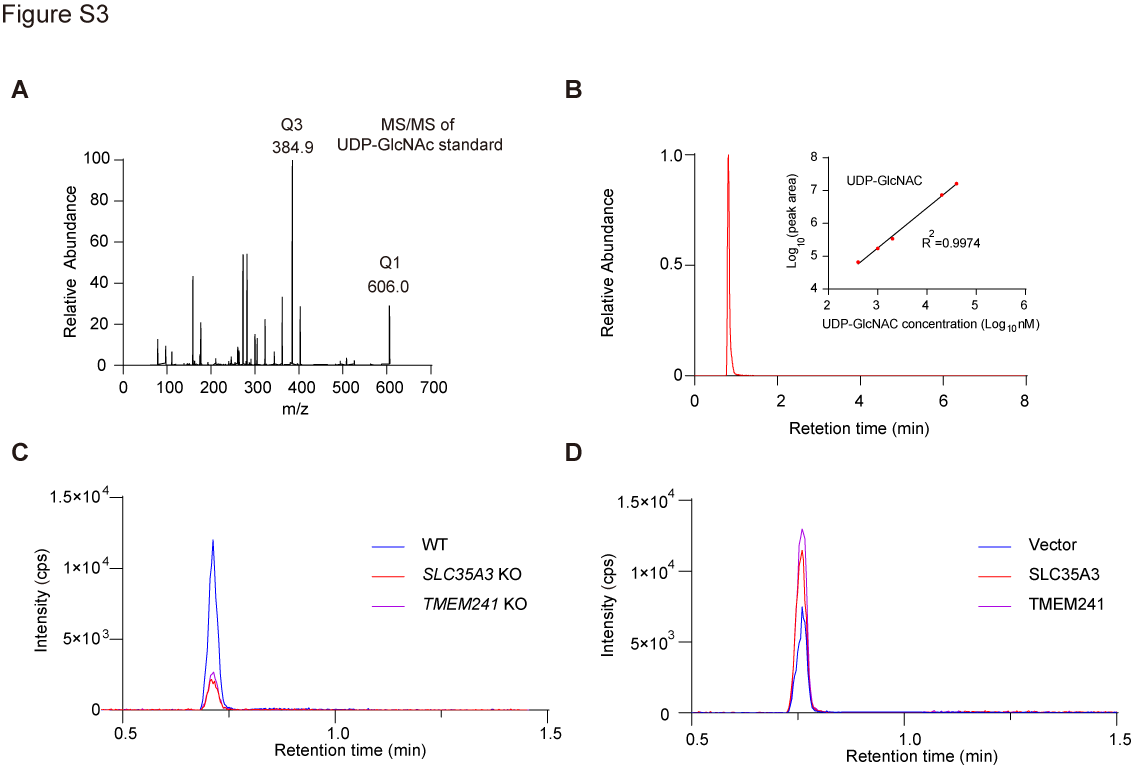
Figure. S4 Detection of UDP-GlcNAc by LC-MRM-MS/MS.**

1. Determination of multiple reaction monitoring (MRM) Q1/Q3 ion pair based on the product ion spectrum of UDP-GlcNAc standard. Q1/Q3(606.0/384.9) was optimized based on MS/MS fragmentation of UDP-GlcNAc standard.
2. The LC–MRM-MS/MS chromatogram of the UDP-GlcNAc standard. The inset shows the linear correlation between the concentration and peak area (R2 = 0.9974).
3. Representative extracted ion chromatograms of UDP-GlcNAc in crude Golgi isolated from control cells (blue), HeLa/*SLC35A3* KO cells (red) and HeLa/*TMEM241* KO cells (purple).
4. Representative extracted ion chromatograms of UDP-GlcNAc in crude Golgi isolated from control (*SLC35A3* KO) cells (blue) and cells overexpressing SLC35A3 (red) and TMEM241 (purple).

**
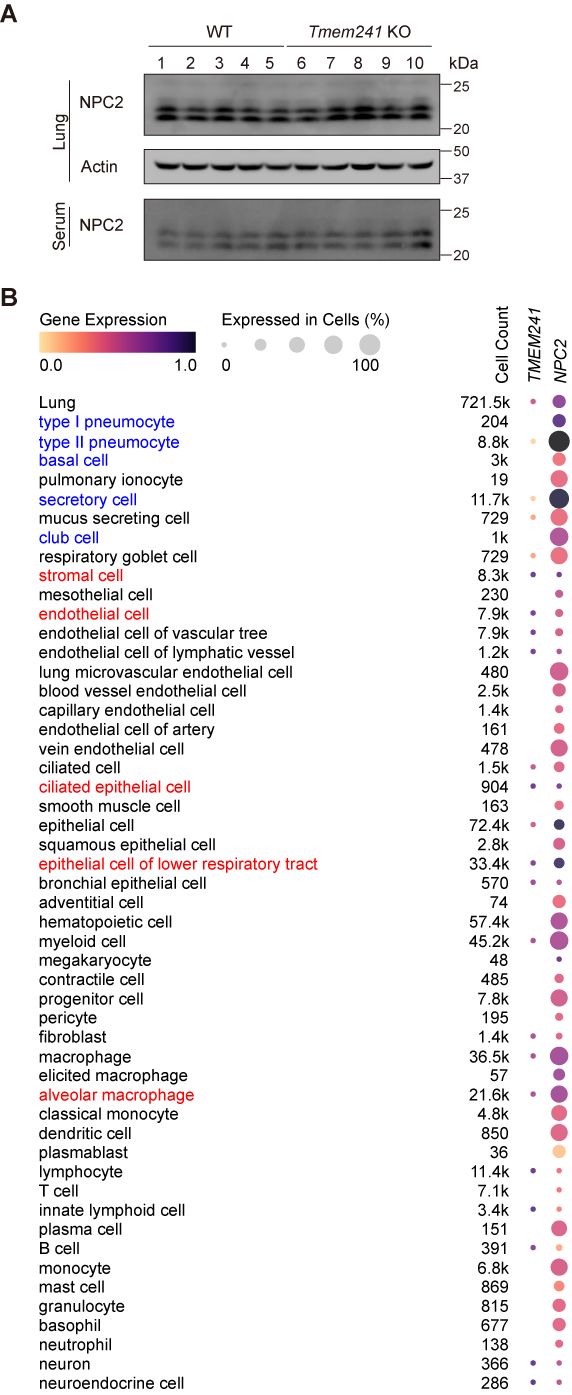
**

**Figure. S5 Analysis the expression of TMEM241 and NPC2.**

(A) The NPC2 proteins in the lung and serum of WT and *Tmem241* KO mice at the age of 8 months (n = 5 per group).

(B) Cell type-specific expression of *TMEM241* and *NPC2* based on available single cell sequencing data. The *TMEM241* and *NPC2* expression analysis in different cell type of normal lung from combined published single-cell lung atlas via cellxgene. Cell types with high co-expression of *TMEM241* and *NPC2* are in red. Those with high expression of *NPC2* and low expression of *TMEM241* are represented in blue.

Supplementary Tables

Table S1. A full list of genes enriched in the cells survived 5 rounds of challenge. Table S1 is shown in a separated Excel file.

Table S2：siRNA sequence for RNA interference

| Gene | Sequence |
| --- | --- |
| *NPC1* | 5’-GCACCAGGTTCTTGACTTA-3’ |
| *NPC2* | 5’-GGATGGAGTTATAAAGGAATT-3’ |
| *TMEM241*-1 | 5’-GACCAGCAATACTTAAACT-3’ |
| *TMEM241*-2 | 5’-GACGCTCATTGGTGGACTT-3’ |
| *EMC3* | 5’-GGATGGATCAACATGACAT-3’ |
| *EMC4* | 5’-CCAGTCGGTTACTTGGACA-3’ |
| *GNPTAB* | 5’-GTACCAACGTTTCTCATGT-3’ |
| *C18ORF8* | 5’-GCATTAAGTTTTCCTTAGA-3’ |
| *WDR91* | 5’-GGACTAACCAGGTTCAAGA-3’ |
| *RAB5C* | 5’-GCACCAGGTTCTTGACTTA-3’ |
| *RAB1A* | 5’-GCACAATTGGTGTGGATTT-3’ |
| *TTC7A* | 5’-GGAGAACCATGCCAAAATA-3’ |
| *TMEM165* | 5’-GCACGGGATTGGCAGTAAT-3’ |
| *SLC35A3* | 5’-GAAGGACCTCGTTATCTAT-3’ |

Table S3：Sequence of primers used in QPCR

| Primer | Sequence |
| --- | --- |
| Q-h-*GAPDH*-F | 5’-AGAAGGCTGGGGCTCATTTG-3’ |
| Q-h-*GAPDH*-R | 5’-AGGGGCCATCCACAGTCTTC-3’ |
| Q-h-*NPC1*-F | 5’-TTACTCAGTTACATAGGGCCATCA-3’ |
| Q-h-*NPC1*-R | 5’-CCTGCGAGAGGGCTAGAAAT-3’ |
| Q-h-*TMEM241*-F | 5’-TGGCCATTCCTGTGTTTCTCA-3’ |
| Q-h-*TMEM241*-R | 5’-GGAGGAGGAGGGCACTACA-3’ |
| Q-h-*EMC3*-F | 5’-TGGGCTTCGGAGCATTTACT-3’ |
| Q-h-*EMC3*-R | 5’-AGCTTCCCACTCTGTCTTGAA-3’ |

Table S3：Sequence of primers used in QPCR

| Primer | Sequence |
| --- | --- |
| Q-h-*GNPTAB*-F | 5’-CCAGTTCGGAGAGGTGGTTC-3’ |
| Q-h-*GNPTAB*-R | 5’-ATCTGTGCCATTCACCCAGG-3’ |
| Q-h-*EMC4*-F | 5’-TGATGGGTTTGGCATTGGCT-3’ |
| Q-h-*EMC4*-R | 5’-AACTCCATTCTCTCAGGGGG-3’ |
| Q-h-*C18ORF8*-F | 5’-AACCCTTGTCCAGCACAACC-3’ |
| Q-h-*C18ORF8*-R | 5’-GCAGCATCTTCAGCATGTCC-3’ |
| Q-h-*WDR91*-F | 5’-CACCACCCCTAAAGACGCTA-3’ |
| Q-h-*WDR91*-R | 5’-TTATCCACCCGGAACCCCTT-3’ |
| Q-h-*RAB5C*-F | 5’-CGGAGCCGCCTTGGAG-3’ |
| Q-h-*RAB5C*-R | 5’-TCCAGCTGTAGTGGTCCAGA-3’ |
| Q-h-*RAB1A*-F | 5’-GCACTGGTTTCCAAAAACGGA-3’ |
| Q-h-*RAB1A*-R | 5’-CCTTCTTCTTAGGTTTGCAGATGA-3’ |
| Q-h-*TTC7A*-F | 5’-CTCCATGCCTTTGCTGGAGA-3’ |
| Q-h-*TTC7A*-R | 5’-ACATGTACTGTGGCGAGAGC-3’ |
| Q-h-*TMEM165*-F | 5’-CGGCCCGGGTCGAGAAAA-3’ |
| Q-h-*TMEM165*-R | 5’-GGCAGCGACAAATGCATGG-3’ |
| Q-h-*NPC2*-F | 5’-GTCCCAGTTCCCTTTCCCATTC-3’ |
| Q-h-*NPC2*-R | 5’-TGCCACTCCACCACCAGTTTTA-3’ |
| Q-m-*18sRNA*-F | 5’-GCAATTATTCCCCATGAACG-3’ |
| Q-m-*18sRNA*-R | 5’-GGCCTCACTAAACCATCCAA-3’ |
| Q-m-*Tmem241*-F | 5’-GACAGGTGACCTCTTCGGTG-3’ |
| Q-m-*Tmem241*-R | 5’-AGCTGTGACGACCTTAGCAC-3’ |
| Q-m-*Il1b*-F | 5’-CCGTGGACCTTCCAGGATGA-3’ |
| Q-m-*Il1b*-R | 5’-GGGAACGTCACACACCAGCA-3’ |
| Q-m-*TNFa*-F | 5’-CATCTTCTCAAAATTCGAGTGACAA-3’ |
| Q-m-*TNFa*-R | 5’-TGGGAGTAGACAAGGTACAACCC-3’ |
| Q-m-*Ccl2*-F | 5’-TACAAGAGGATCACCAGCAGC-3’ |
| Q-m-*Ccl2*-R | 5’-ACCTTAGGGCAGATGCAGTT-3’ |
| Q-m-*Ccl5*-F | 5’-TGCTGCTTTGCCTACCTCTC-3’ |
| Q-m-*Ccl5*-R | 5’-TCTTCTCTGGGTTGGCACAC-3’ |

Table S3：Sequence of primers used in QPCR

| Primer | Sequence |
| --- | --- |
| Q-m-*Cxcl10*-F | 5’-ATGACGGGCCAGTGAGAATG-3’ |
| Q-m-*Cxcl10*-R | 5’-ATGATCTCAACACGTGGGCA-3’ |
